# Laparoscopic assisted right hemicolectomy for caecal volvulus

**DOI:** 10.1186/1749-7922-3-4

**Published:** 2008-01-21

**Authors:** Michael D Kelly, John Bunni, Anne M Pullyblank

**Affiliations:** 1 Department of Upper GI, Frenchay Hospital, Bristol, UK; 2 Department of Colorectal Surgery, Frenchay Hospital, Bristol, UK

## Abstract

A case of acute caecal volvulus is presented where laparoscopy was used to avoid a major laparotomy. A technique is described for safe deflation of the caecum, which allowed a small incision for resection and anastomosis.

## Introduction

Acute caecal volvulus is an uncommon cause of the acute abdomen and the diagnosis may not be immediately apparent. It presents with abdominal pain, vomiting and abdominal distension. Laparoscopy is still somewhat controversial for bowel obstruction and the case reported herein is presented to show the advantage of laparoscopy for caecal volvulus.

## Case presentation

A 57-year-old woman was admitted to hospital with several days of abdominal pain, vomiting, abdominal distension and constipation. She had suffered for two years with intermittent bouts of colicky abdominal pain and loose bowels, which previously had been diagnosed as irritable bowel syndrome, although no investigations had been done.

**Figure 1:**
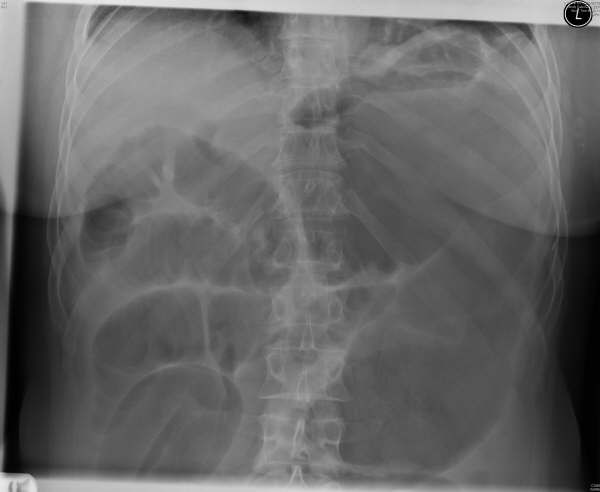
Plain abdominal x-ray showing small bowel obstruction with dilated large bowel in the left upper quadrant.

**Figure 2:**
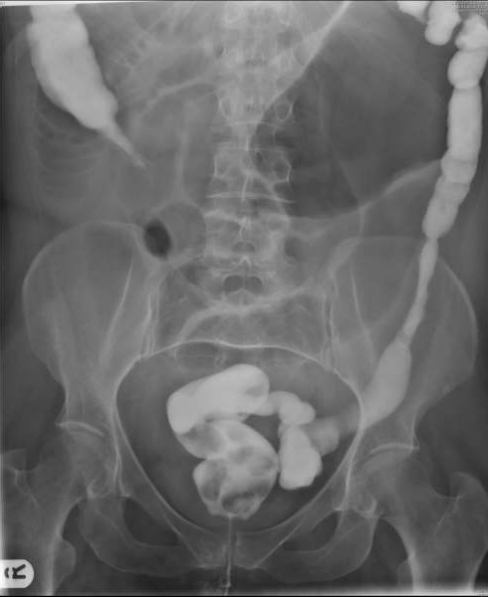
Contrast enema showing abrupt cut off in the ascending colon due to caecal volvulus.

On examination she was febrile and tachycardic with abdominal distension and tenderness. Blood tests showed a raised white cell count and abdominal x-rays showed dilated loops of small bowel with a dilated loop of large bowel in the left upper quadrant (figure 1). Although caecal volvulus was suspected, a Gastrografin enema (Schering AG, Germany) was done to confirm the diagnosis while operation was arranged (figure 2).

**Figure 3:**
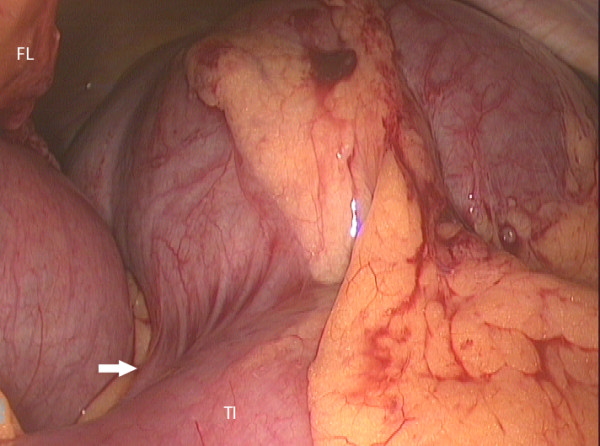
Laparoscopic view of the left upper quadrant showing the distended caecum due to a 180° clockwise rotation of right colon (Falciform ligament – FL, terminal ileum – TI, arrow points to the base of the appendix).

**Figure 4:**
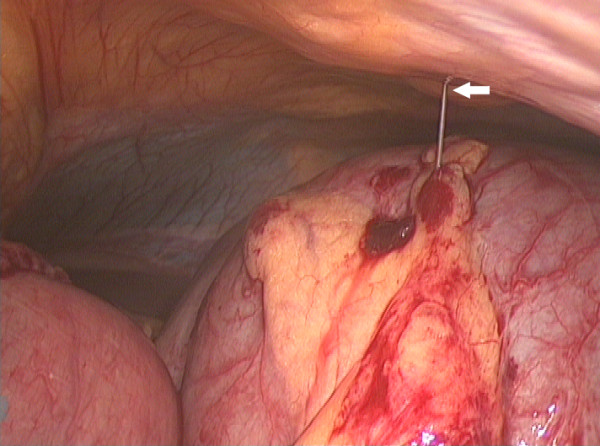
25G spinal needle inserted through anterior abdominal wall into the caecum (arrow).

**Figure 5:**
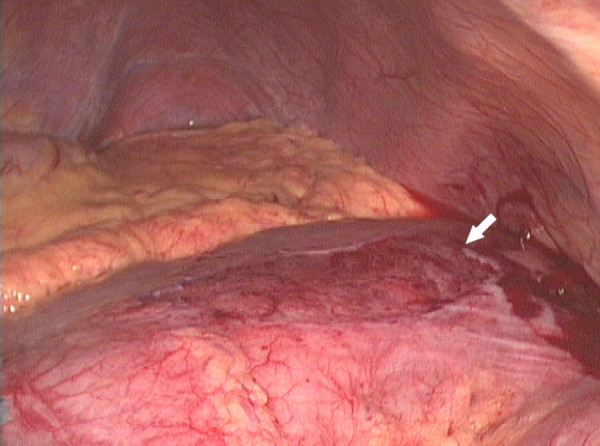
Deflated caecum prior to "untwisting". A large serosal tear is evident (arrow).

The pneumoperitoneum was carefully established after open insertion of a 10 mm cannula through the umbilicus and subsequently two 5 mm ports were added. The tense, distended caecum was visible in the left upper quadrant and there was dilated small bowel and collapsed colon (figure 3). A 25G spinal needle was inserted through the anterior abdominal wall into the caecum. It was connected to a 10 ml syringe barrel and suction tubing, which was connected to wall suction (figure 4). The caecum was rapidly deflated and it was viable although there was a large serosal tear (figure 5). The caecum was flipped back to be in a more normal position and the umbilical port side was enlarged. The caecum and terminal ileum were delivered and a limited right hemicolectomy was performed with end-to-end anastamosis of terminal ileum to ascending colon with interrupted 3-0 Vicryl (Ethicon, New Jersey, USA). The patient made an uneventful recovery and was discharged home on day 5.

## Discussion

Caecal volvulus is a rare cause of the acute abdomen and accounts for approximately 25% of cases of acute colonic volvulus [1,2]. It can occur when the ascending colon, caecum and terminal ileum are abnormally mobile, which represents failure, *in utero*, of complete rotation, descent and fixation of the right colon.

Patients with acute caecal volvulus present with features of strangulating distal small bowel obstruction. Abdominal x-rays may be diagnostic, however if further imaging is felt necessary then computed tomography (CT) or water-soluble contrast enema should be diagnostic.

Operative management is needed for acute volvulus with signs of strangulation. Resection is obviously mandatory when the caecum is gangrenous. When the caecum is viable then caecostomy, caecopexy or resection is acceptable depending on the condition of the patient.

Caecostomy, either formal or tube, may be used after untwisting, for decompression and fixation in very poor risk patients as a life saving measure.

Caecopexy involves untwisting of the caecum and fixation by sutures or use of a peritoneal flap, however, it has a high recurrence rate of up to 20% [1]. It may be combined with tube caecostomy.

Resection is the procedure of choice as it excludes the possibility of recurrence. Primary anastomosis is optimal although there may be cases where it is felt safest to bring out an end ileostomy. One study suggested that primary anastomosis can be performed in the presence of a gangrenous caecum without increased risk of anastomotic leak in otherwise fit patients [3].

Laparoscopic surgery for the acute abdomen has been shown to be beneficial and many conditions once considered "off limits" are now being routinely managed laparoscopically. Laparoscopy for acute caecal volvulus had not been widely reported and this is probably due to the rarity of the condition and perceived difficulties with using laparoscopic techniques. As the bowel may be very distended there is the possibility of injury when establishing the pneumoperitoneum and an open technique using strong ventral traction on the abdominal wall is advisable. Also the bowel distension may make it difficult to get good views within the peritoneal cavity. As the caecum is very tense it is not possible to handle it until it has been aspirated and we found transabdominal aspiration as described herein to be useful. As the caecum is completely mobile it can easily be delivered via the enlarged umbilical port site for resection and hand anastomosis.

Laparoscopic colectomy is well established and it is standard practice to divide the mesenteric vessels intracorporeally (and sometimes the bowel) before the laparoscopy is discontinued and the colon is delivered through an enlarged port site. In this case, there was no need to do this as the colon was mobile enough to be easily delivered through the enlarged umbilical port site without dividing the vessels laparoscopically and, as this was a benign condition, radical resection of the mesentery, vessels and lymphatics was not needed.

Compared to our experience with open surgery for acute caecal volvulus we found that laparoscopy and aspiration facilitated a safe operation and rapid recovery.
